# Editorial: Women in molecular and cellular oncology, volume II: 2022

**DOI:** 10.3389/fonc.2023.1272365

**Published:** 2023-09-01

**Authors:** Shilpa S. Dhar

**Affiliations:** Department of Molecular & Cellular Oncology, The University of Texas M. D. Anderson Cancer Center, Houston, TX, United States

**Keywords:** women, molecular oncology, therapeutic targets, cancer, tumor microenvironment

The COVID-19 emergency has worsened existing inequalities, especially those related to sex and gender. Women have been disproportionately affected, experiencing increased violence and caregiving responsibilities during lockdowns, economic hardships, disrupted health services, and school closures (1). Unfortunately, progress toward gender equality has been eroded by early 2023 (2, 3). Therefore, it’s crucial to prioritize gender in addressing global challenges to create a more equitable world. Women in oncology, including clinical trials, have made significant contributions, advancing cancer research and treatment on par with men. Despite facing historical biases, they excel in molecular and cellular oncology and foster diverse collaborations. Empowering women in this field requires promoting mentorship, supportive work environments, gender equality in funding, and leadership roles. By embracing the potential of all scientists, we can accelerate the fight against cancer and improve treatments. This article aims to highlight women scientists’ diverse research in oncology. This impressive Research Topic highlights 14 distinguished articles on various forms of cancer, all of which were authored by remarkable women.

Lung cancer is the top cause of cancer deaths; it surpasses the combined deaths from breast, prostate, and pancreatic cancers (4). Liu et al. investigated the role of m6A demethylase ALKBH5 in non-small-cell lung cancer-derived cancer stem-like cells (CSCs). The study revealed that ALKBH5 is highly expressed in lung CSCs, positively correlated with p53. Knocking down ALKBH5 reduced stem markers and inhibited stemness, while p53 knockdown decreased ALKBH5 expression and malignancies. This p53/ALKBH5 axis may be a potential therapeutic target for NSCLC.

Moreover, CDK7 was significantly overexpressed in squamous cell carcinomas compared to adenocarcinomas, with high CDK7 levels linked to worse overall and disease-free survival in NSCLC patients. Kuempers et al. proposed CDK7 as a promising prognostic biomarker for NSCLC and a potential target for future anticancer therapies.

Furthermore, Owczarek et al. investigated that The CAR protein, highly expressed in lung cancer, promotes tumor growth through increased cell adhesion and enhanced b1 integrin activity, leading to invasion in 3D models. CAR forms a complex with focal adhesion proteins, activating the GTPase Rap1, which mediates enhanced integrin activation and potentially contributes to lung cancer metastasis.

Targeted therapies are crucial for lung cancer with low survival rates. Ion channels called Acid sensors like ion channels (ASICs) are promising targets that detect changes in pH levels within the tumor microenvironment. Several types of ASICs and degenerin/epithelial Na+ channel (DEG/ENaC) and ENaC subunits can form different channels, influencing how cells respond to stimuli. Sudarikova et al. demonstrated that Mambalgin-2 inhibits the growth and migration of lung adenocarcinoma cells by inducing G2/M cell cycle arrest and apoptosis. It targets ASIC1a/α-ENaC/ץ-ENaC heterotrimeric channels, bypassing resistance mechanisms in bulky tumors, thus holding potential for novel channel-targeting anticancer drugs.

Malignant melanoma, a highly aggressive skin malignancy causing significant global mortality, exhibits an intriguing duality. While it contributes to numerous deaths, it also sparks a robust anti-tumor immune response, one of the most immunogenic cancers. Kwiatkowska et al. has compiled data showing that the transcription factor Yin Yang 1 (YY1) is key in cancer progression. It controls genes related to cell death, immune response, and metastasis. It also affects melanoma survival, proliferation, and stem cell-related transcription factors. Focusing on YY1 and its pathways can lead to novel therapeutic strategies, improving melanoma treatment. More research is needed to determine its specific role in pathogenesis, progression, and drug resistance for potential therapeutic breakthroughs.

Another exciting review was presented by Popovic and Tartare-Decket about the extracellular matrix (ECM) as a critical component for tissue homeostasis. Changes in the ECM structure of melanoma tumors increase stiffness, promoting disease progression and poor survival rates. Melanoma cells interact with the ECM through receptors, secreted factors, or enzymes. Stromal fibroblasts deposit and remodel the ECM, enabling interactions with melanoma cells. Understanding ECM properties is critical to understanding melanoma progression and resistance. Targeting ECM abnormalities with therapies has the potential to improve treatment outcomes.

Prasuhn et al. describes a case report of a patient with malignant uveal melanoma and exudative retinal detachment, treated with plaque brachytherapy resulting in tumor regression. After one year, a ring-shaped recurrence with extraocular extension appeared, necessitating enucleation. Genetic analyses revealed GNAQ and SF3B1 mutations not previously reported in ring melanoma. Ring melanoma is a rare and aggressive variant of uveal melanoma with limited treatment options. Regular follow-up and genetic testing are essential to monitor disease progression and identify potential therapeutic targets. This case sheds light on the genetic background of ring melanoma, contributing to the development of personalized treatment strategies.

Prostate cancer (PCa) is a significant health concern for men, leading to numerous diagnoses and cancer-related deaths. Despite successful treatments, recurrence remains a challenge. Garcı´a-Vargas et al. explored the role of HLA-B-associated transcript 1 (BAT1) in PCa. BAT1 was found to be differentially expressed in patients with varying Gleason scores. This report suggests that BAT1 expression plays a role in prostate cancer. Reducing BAT1 increases cell migration and inflammation, while overexpression decreases these processes. By understanding BAT1’s role, one can develop better treatments for recurrent PCa.

Erfanparast et al. presented a detailed review of Oral cancer and non-coding RNAs (ncRNAs). Despite advancements in treatment, the five-year survival rate remains low, and the molecular mechanisms underlying oral cancer development are not fully understood. Noncoding RNAs (ncRNAs), including microRNAs, long ncRNAs, and circular RNAs, play crucial roles in cancer cell development, including cell death processes such as apoptosis and autophagy. Understanding the regulatory relationships between ncRNAs and cell death pathways is critical in developing targeted therapies for oral cancer. Manipulating the expression of apoptosis-regulating ncRNAs may represent a strategy to combat carcinogenesis and enhance the response to chemotherapy and radiotherapy. Identifying and profiling ncRNAs in clinical samples can aid in predicting patient responses to treatments and designing personalized therapeutic strategies for oral cancer.

Gliomas, the most common brain tumors, are known for their poor prognosis due to tumor cell migration and invasion in the brain. Peris-Celda et al. found that high levels of SuFu, a molecular mediator in this pathway, were associated with increased dissemination patterns in glioma patients. Further experiments showed that SuFu overexpression increased cancer stemness properties and a migratory phenotype in Glioblastoma Cancer Stem Cells (GB CSCs). Further research is needed to confirm its role as a mediator of tumor spread and develop effective blocking agents for improved patient outcomes.

Resistance training (RT) in tumor-bearing mice can prevent cancer-induced muscle atrophy. Testa et al. investigated the role of signal transducers and activators of transcription (STAT3) in mediating muscle atrophy and the effects of RT on its activation. Resistance training can prevent muscle atrophy caused by cancer in mice by reducing STAT3 activation, downregulating key genes and proteins, and improving strength and locomotor capacity. This also leads to improved quality of life for cancer patients.

Sapochnik et al. have suggested that targeting Nrf2 and its associated proteins could be a promising therapeutic approach for treating Kaposi’s sarcoma (KS), a frequently occurring tumor in individuals with AIDS.

Cyran and Zhitkovich conducted a detailed analysis of how heat-shock response (HSR) is activated to manage high levels of proteotoxic stress experienced by cancer cells because of gene mutations and dysregulation. This results in an increase in heat shock proteins (HSPs) and HSF1. High levels of HSR components are linked to drug resistance and poor clinical outcomes in cancer. Targeting the HSR pathway is a promising approach for treating human malignancies as it is a common vulnerability in cancer cells.

Fayzullina et al. described Azvudine (FNC) as a novel cytidine analog with antiviral and anticancer properties. Its inhibitory effects on RNA viruses and retroviruses, including HIV and SARS-COV-2, make it a promising antiviral agent. FNC can impede malignant cell growth and induce apoptosis as an anticancer drug. Further research is needed to understand its precise mechanisms of action. As a group of female scientists who have contributed 14 exciting articles to the Women in Molecular and Cancer Oncology 2022 edition, we strongly advocate for gender equality in oncology research. By providing more opportunities for women to conduct research, we can enhance the quality and impact of our work, ultimately benefiting all cancer patients. Our goal is to promote progress toward gender equality in the field, and we are dedicated to making this a reality.

## Author contributions

SD: Conceptualization, Visualization, Writing – original draft, Writing – review & editing.
